# *Bordetella pertussis* Clones Identified by Multilocus Variable-Number Tandem-Repeat Analysis

**DOI:** 10.3201/eid1602.081707

**Published:** 2010-02

**Authors:** Jacob Kurniawan, Ram P. Maharjan, Wai-Fong Chan, Peter R. Reeves, Vitali Sintchenko, Gwendolyn L. Gilbert, Frits R. Mooi, Ruiting Lan

**Affiliations:** University of New South Wales, Sydney, New South Wales, Australia (J. Kurniawan, R.P. Maharjan, W.-F. Chan, R. Lan); University of Sydney, Sydney (P.R. Reeves, V. Sintchenko, G.L. Gilbert); Westmead Hospital, Westmead, New South Wales, Australia (V. Sintchenko, G.L. Gilbert); National Institute for Public Health and the Environment, Bilthoven, the Netherlands (F.R. Mooi).

**Keywords:** multilocus variable-number tandem-repeat analysis, acellular vaccine antigenic variation, Bordetella pertussis, *g*lobal epidemiology, bacteria, dispatch

## Abstract

Multilocus variable-number tandem-repeat analysis (MLVA) of 316 *Bordetella pertussis* isolates collected over 40 years from Australia and 3 other continents identified 66 MLVA types (MTs), including 6 predominant MTs. Typing of genes encoding acellular vaccine antigens showed changes that may be vaccine driven in 2 MTs prevalent in Australia.

Despite longstanding vaccination programs, pertussis remains endemic to many industrialized countries, including Australia, Canada, Italy, Japan, the Netherlands, Switzerland, and the United States, all of which have reported recent increases in incidence (*1*). Although pertussis is classically a disease of infants and children, this increase has been mainly among adults and adolescents (*2*,*3*). Factors contributing to pertussis resurgence remain unclear, but possible causes are waning immunity, suboptimal vaccine coverage, improved surveillance and diagnosis, the switch from whole cell vaccine (WCV) to acellular vaccine (ACV), and adaptation of circulating *Bordetella pertussis* strains (*4*–*9*). To determine the global epidemiology of pertussis, we analyzed an international collection of *B. pertussis* isolates collected mainly over the past 40 years.

## The Study

We used 8 variable-number tandem-repeats, including 6 from Schouls et al. (*10*), to develop a multiplex PCR multilocus variable-number tandem-repeats analysis (MLVA) assay (Table 1; Technical Appendix) and used it to characterize 316 *B. pertussis* isolates from 12 countries on 4 continents, including 208 isolates from Australia and 87 isolates representative of common pulsed field gel electrophoresis types from Canada, Japan, Finland, and the United States (complete list available from authors). The Simpson index of diversity (*D*) ranged from 0.02 to 0.73 per locus with a combined *D* of 0.911 (Table 2). The isolates were resolved into 66 MLVA types (MTs) (complete list available from authors). Thirty-five MTs were represented by single isolates, including 15 of 208 isolates from Australia and 10 of 49 isolates from Japan. Thirty-seven MTs were previously found in Europe (*10*,*11*) and 27 were novel. Fourteen MTs were found in >2 countries or regions.

**Table 1:** Primers used in study of *Bordetella pertussis* clones Identified by multilocus variable-number tandem-repeat analysis

Primer name	Sequence, 5′ → 3′	Genome coordinates*	Mix	Concentration,† μM	Reference
BP-VNTR1-DF	VIC-CCTGGCGGCGGGAGACGTGGTGGTG	2194507	1	0.13	(*10*)
BP-VNTR1-DR	AAAATTGCGGCATGTGGGCTGACTCTGA	2194862	1		(*10*)
BP-VNTR2-BF	VIC-CGCGCCGCCTACGACCGCTATGG	2647550	2	0.08	(*10*)
BP-VNTR2-BR	CCCGCGCCGAAGATCTCGCCAAAGATAT	2647412	2		(*10*)
BP-VNTR3-BF	FAM-GCCTCGGCGAAATTGCTGAAC	2591464	2	0.23	(*10*)
BP-VNTR3-BR	GCGGGCGAGGAAACGCCCGAGACC	2591350	2		(*10*)
BP-VNTR4-CF	NED-CGTGCCCTGCGCCTGGACCTG	185211	2	0.08	(*10*)
BP-VNTR4-BR	GCCGCTGCTCGACGCCAGGGACAA	185000	2		(*10*)
BP-VNTR5-BF	PET-GAAGCCGGCCCACCCGAGCTCCAGGCTCTT	1005290	1	0.06	(*10*)
BP-VNTR5-BR	TGCCGGGTTTCGGCATCTCGATGGGATACG	1005177	1		(*10*)
BP-VNTR6-EF	FAM-CCAACGGCGGTCTGCTGGGTGGTC	2099525	1	0.06	(*10*)
BP-VNTR6-FR	CGCCGCCCGCTGCGCCGCTACC	2099315	1		(*10*)
VNTR7F2	PET-ATCAGGAAACCCACCACCACGCCGG	124402	2	0.08	This study
VNTR7R2	GTCACCAGCCCGCAGTACTGGCG	124585	2		This study
VNTR8F2	NED-TGGGTGTCTCCGTGATAGTGAGCACTTACAC	444776	1	0.19	This study
VNTR8R2	CTGGCGCAAAAACAGTAAGCCCGCACG	444981	1		This study

*Based on genome sequence position of the Tohama I strain; VNTR, variable-number tandem-repeat. †Concentrations listed are for forward and reverse primers separately.

**Table 2:** Diversity of variable-number tandem-repeat analysis loci *Bordetella pertussis* isolates*

Locus	No. repeats	Global (this study)			Australia (this study)			The Netherlands (*10*)	
		No. alleles	*D*		No. alleles	*D*		No. alleles	*D*
VNTR1	2–12	6	0.58		4	0.63		7	0.26
VNTR2	2–5	4	0.02		2	0.01		3	N/A
VNTR3a	2–8	4	0.40		4	0.43		10	0.18
VNTR3b	0–10	5	0.21		5	0.21		4	0.15
VNTR4	2–9	7	0.34		5	0.24		8	0.21
VNTR5	3–9	6	0.20		4	0.19		7	0.18
VNTR6	2–11	8	0.72		5	0.70		8	0.60
VNTR7	3–4	2	0.01		1	0.00		NA	NA
VNTR8	2–4	3	0.16		2	0.03		NA	NA

*D, Simpson index of diversity; VNTR, variable-number tandem-repeat; NA, not applicable.

**Figure 1:**
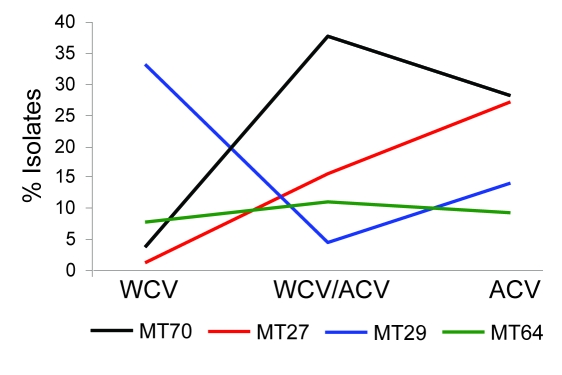
Temporal trends of predominant multilocus variable-number tandem-repeat analysis (MLVA) types in Australia. Isolates of 4 major MLVA types (MT70, MT27, MT29, and MT64) obtained in Australia were divided into 3 periods: whole cell vaccine (WCV) (before 1997), transition from WCV to acellular vaccine (ACV) (1997–1999), and ACV (2000 onward).

The 208 isolates from Australia were grouped into 37 MTs, of which the 4 most prevalent represented 65.4% of the isolates: MT27, 13.5%, including 1 isolate from 1973 and the others from the 1990s to 2008; MT29, 21.6%, observed since 1972; MT70, 21.2%, 1996–2005, mostly since ACV introduction in 1997; and MT64, 9.1%, during 1989–2002. Prevalence trends of the 4 most common MTs were analyzed for 3 periods determined by vaccine type(s) in use: WCV (prior to 1997), the transition period of both WCV and ACV (1997–1999), and ACV only (2000 onward) (Figure 1). MT64 prevalence was steady over time. MT29 decreased while MT27 and MT70 increased. Trends in Australia for MT27 and MT29 were similar to those observed in the United Kingdom (*11*) and the Netherlands (*10*). D values were 0.86, 0.83, and 0.83 for WCV, transition, and ACV periods, respectively. This slight decrease in genetic diversity might indicate expansion of clones that are better adapted to ACV-induced immunity.

We typed 5 genes, the products of which are used in ACVs (*prn*, *ptxA, fim2, fim3,* and *fhaB*), using the method of Chan et al. (*12*) to assess the effect of the switch from WCV to ACV on prevalence of the 4 predominant MTs (MT27, MT29, MT64, and MT70) in Australia. Isolates from these MTs have the same *ptxA1* and *fhaB1* alleles but vary in the other 3 genes investigated (complete list available from authors). The predominant ACV used in Australia is from GlaxoSmithKline (GSK) (Research Triangle Park, NC, USA), which contains pertussis toxoid, filamentous hemagglutinin, and pertactin but no fimbriae (FIMs). The strain used for GSK ACV contains the alleles *prn1*, *ptxA2,* and *fhaB1* (*10*,*13*). However, ACV from Sanofi-Aventis (Pasteur, Lyon, France), which contains FIM2+3 in addition to pertussis toxoid, pertactin, and filamentous hemagglutinin with unknown allele types, is also licensed in Australia, complicating interpretation of variation in *fim* genes. On the basis of their frequencies and late appearance, *fim2-2* and *fim3-B* are not likely to be the vaccine alleles. A significant increase (p<0.005) of *prn2* (36% vs. 3%)*,* *fim2-2* (34% vs. 8%), and *fim3-B* (24% vs. 0%) was observed in the ACV period in comparison to the WCV period.

This increase of allelic frequency is better reflected in changes in antigenic profiles. MT27 has 3 profiles (*prn1, fim2*-1, *fim3*-A*; prn2, fim2*-1*, fim3*-A*;* and *prn2, fim2*-1*, fim3*-B). The first profile was seen once in the WCV period, whereas the other 2 first appeared in the WCV/ACV transition period and increased in frequency in the ACV period; the third profile, which differed by 2 alleles from the first, was more frequent. The appearance of *prn2* in the second profile and additional change from *fim3-A* to *fim3-B* in the third represent increases in prevalence of alleles absent from ACV. MT29 also has 3 profiles ([*prn1, prn2,* or *prn3*], *fim2*-1, *fim3*-A), which differ in *prn* only. Most MT29 isolates carry *prn3,* and the profile is prevalent in both WCV and ACV periods, with no obvious increase in non-ACV alleles.

MT70 and MT64 both have uniform allelic profiles (*prn1*, *fim2*-2, *fim3*-A and *prn1*, *fim2*-1, *fim3*-A, respectively)**.** However, MT70 (with *fim2-2,* not likely to be in ACV) increased significantly over the study period while MT64 with all alleles likely to be in ACV remained steady. Overall, the frequency of MT27 and MT70, with non-ACV alleles, increased significantly (p<0.0001) and correlated with the introduction of ACV, suggesting that antigenic changes could be driven by selection pressure.

The 2 MTs predominant in Australia were also prevalent in other countries and possibly have a global distribution. MT27 (18% of isolates) was found in 8 countries and MT29 (17% isolates) in 5. However, absence of an MT in a country might result from the small samples used**.** MT27 and MT29 were the most common types in the Netherlands (*10*) and the United Kingdom (*11*). MT10, MT64, MT70, MT84, and MT186 were also relatively common. MT10 and MT186 were found predominantly in Japan, although each had been found elsewhere, in China (1957) and Hong Kong (2002), respectively. MT64 was predominantly from Australia with 1 isolate from Japan, and MT70 was only found in Australia. However, all of these frequent MTs (except MT186) have been observed before. MT10 was frequent in the United Kingdom in the prepertussis vaccine era, while MT70 was common during 1998–2001 (*11*).

Nine isolates, including Tohama I, indentified in samples of pertussis strains collected during the 1920s–1950s from 5 countries (China, France, Japan, United Kingdom, and United States) were distributed among 7 MTs: MT10, MT12, MT75, MT83, MT127, MT205, and MT206, 2 of which were also represented among recent strains: MT10, 6 isolates from Japan 1989–2007; and MT75, 1 isolate from France in 1993. The remaining 5 MTs were either unique or shared only among the 9 early isolates.

**Figure 2:**
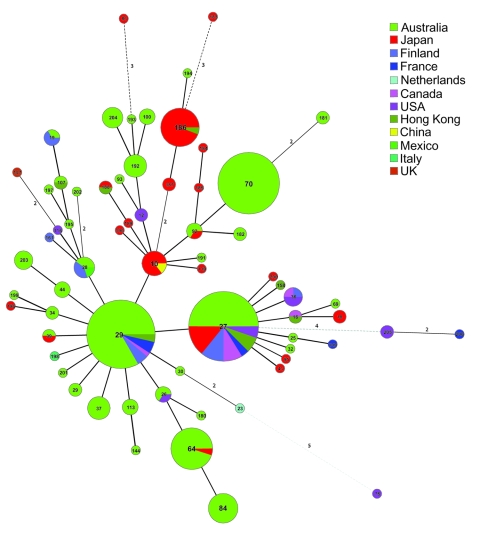
Minimum spanning tree (MST) of multilocus variable-number tandem-repeat analysis (MLVA types for global *Bordetella pertussis* isolates. The MST produced in Bionumerics (Applied Maths, Kortrijk, Belgium) used categorical coefficient and the eBURST priority rule of the highest number of single-locus changes for the clustering. Each circle represents an MLVA type with the type number in the circle. Thick lines, types differing by a single MLVA locus; thin lines, double-locus variants; dotted lines, 2 types differing by >2 MLVA loci. The size of the circle reflects the number of isolates with a given MLVA type. The color codes for country of origin are shown, and pie charts within a circle are used to indicate the proportion of isolates.

MLVA data were used to construct a minimum spanning tree (MST) (Figure 2). The 66 MTs were grouped into 2 clonal complexes and 9 singletons. Most MTs (54 of 66) belong to 1 clonal complex and 3 (MT186, MT187, and MT194) belong to another. Relationships between singletons with multiple allelic differences are not robust because they can be connected to other nodes equally. Thus, the MST cannot be rooted to infer the direction of change. Two internationally predominant MTs (27 and 29) are closely related with 1 allele difference. MT10, prevalent in Japan, is also closely related to MT29, with 1 allele difference. MT29, first isolated in the prevaccine era in the United Kingdom (*11*), has the highest number of SLVs and was found over 4 continents, which suggests that it arose early. Because MT10 and MT27 have a high frequency of SLVs, both likely emerged quite early. MT10 was isolated as early as 1957 in China and MT27 in 1950 in the Netherlands (*10*). Two high-frequency MTs (MT64 and MT70) were found in Australia only recently, with few SLVs, and may have contributed to the resurgence of pertussis in Australia.

## Conclusions

Analysis of 208 isolates from Australia and representative isolates of common pulsed-field gel electrophoresis types from Canada, Japan, Finland, and the United States identified 6 predominant MTs (clones). Two (MT27 and MT29) were distributed worldwide, while 4 (MT10, MT64, MT70 and MT186) predominated in specific countries. Several MTs have persisted over long periods, including 3 that have circulated for at least half a century. Typing of genes encoding ACV antigens showed that use of ACV may have driven antigenic changes of 2 MTs now predominant in Australia.

## Supplementary Material

Technical Appendix Bordetella pertussis Clones Identified by Multilocus Variable-Number Tandem-Repeat Analysis
