# Supplementary material for: Bordetella pertussis Clones Identified by Multilocus Variable-Number Tandem-Repeat Analysis
Source: Emerg Infect Dis. 2010 Feb;16(2):297–300. doi: 10.3201/eid1602.081707 (PMC2957989; doi:10.3201/eid1602.081707)
Supplement: Technical Appendix — Bordetella pertussis Clones Identified by Multilocus Variable-Number Tandem-Repeat Analysis [file 08-1707_Techapp-s1.pdf]

Bordetella pertussis Clones Identified by Multilocus Variable-Number Tandem-Repeat Analysis

Technical Appendix

Isolates Used in This Study

A complete list of the isolates used in this study is available from the author. The majority were recent isolates: 208 from Australia (1970s–2008), 49 from Japan (1989–2007), excluding the Tohama I strain, with 36 being representatives from the 48 pulsed field gel electrophoresis types identified previously by Kodama et al. (*1*), 15 from Finland (1991–2006), 11 from Hong Kong (2002–2006), 8 from France (1993– 2007), 12 from the United States (2002–2006), 8 from Canada (1994–2005) and 1 each from the Netherlands (1992), Italy (1994), China (1957), United Kingdom (1920), and Mexico (year of isolation unknown). The isolates from Canada, Finland, and the United States were representatives of most frequent pulsed field gel electrophoresis types from the respective countries and therefore represent the prevalent strains currently circulating in these countries. Nine strains, including Tohama I, were isolated before 1960, one each from China, France, Japan, and the United Kingdom, and 5 from the United States.

Multilocus Variable-Number Tandem Repeat Analysis

We explored new variable-number tandem repeat (VNTR) analysis loci in addition to the 6 (VNTRs 1–6) previously identified by Schouls et al. (*2*). Forty-one potential VNTR loci from the *B. pertussis* Tohama I genome (*3*) were identified and screened on a panel of 18 *B. pertussis* isolates selected on the time and locality of isolation (Supplementary Table 1). Two new VNTR loci named VNTR7 and VNTR8 were found to be variable in the 18 isolates tested with 2 and 3 alleles, respectively. They were added to the initial set of 6 to develop an 8-plex PCR reaction. Because no conditions satisfied the simultaneous amplification of all 8 products consistently, the primers were divided between two 4-plex PCR assays with VNTR 1, 5, 6, and 8 in 1 reaction and VNTR 2, 3, 4, and 7 in the other. The thermal cycling parameters for the 2 assays were identical, except that the annealing temperature was 68°C for the first and 60°C for the second 4-plex PCR.

The primers and dye labels are listed in Table 1 in the main text. Each 4-plex PCR was performed in 20-µL volumes (100 µM dNTPs, 20 mM Tris-HCl, 10 mM $(NH_4)_2SO_4$, 10 mM KCl, 2 mM $MgSO_4$, 0.1% Triton X-100, 0.25 units/µL Taq DNA polymerase [New England Biolabs, Ipswich, MA, USA]). The thermal cycling parameters were an initial denaturation step at 96°C for 3 min, followed by 40 cycles of 96°C for 20 s, 68°C for 30 s for mix 1 or 60°C for 30 s for mix 2, 72°C for 1 min and 30 s, and a final extension step of 72°C for 20 min. Final PCR products for mix 1 and mix 2 were diluted 1:100 and 1:200, respectively before the fragments were separated on an ABI 3700 DNA sequencer. All VNTRs were amplified consistently.

For each VNTR, the size of the PCR product was converted to number of repeat units as alleles. For consistency, we used the same MLVA type numbering scheme as for the 5-VNTR loci (excluding VNTRs 2, 7, and 8) (*2*). When a 5-locus type is subdivided, because of differences at one or more of the 3 additional loci, the most frequent subtype takes the type number and any other subtypes were named by adding a letter to the type number. Only 1 MLVA type has a subtype in the 8-locus scheme. MLVA type 29b (2 isolates, profile 8-3-7-0-7-6-10-3-3) is a subtype of MLVA type 29 (profile 8-3-7-0-7-6-9-3-3). Therefore, MLVA types based on 5 VNTR loci are compatible with those based on 8 loci, which allows comparison with previous typing data. Although the extra 3 VNTRs do not contribute greatly to discriminatory power, their inclusion is useful because they act as confirmatory markers for some genotype divisions. MLVA type is abbreviated as MT in the main text.

Bioinformatics

A minimum spanning tree (MST) was constructed by using Bionumerics (Applied Maths, Kortrijk, Belgium) based on categorical coefficient and the eBURST (*4*) priority rule of the highest number of single-locus changes for the clustering. A clonal complex was defined as consisting of isolates that were single locus variants (SLVs) of the predominant MLVA type (*4*). The Simpson's index of diversity (D value) was calculated by using an in-house program, the MLEECOMP package (*5*).

References

1. Kodama A, Kamachi K, Horiuchi Y, Konda T, Arakawa Y. Antigenic divergence suggested by correlation between antigenic variation and pulsed-field gel electrophoresis profiles of *Bordetella*

pertussis isolates in Japan. J Clin Microbiol. 2004;42:5453–7. PubMed DOI: 10.1128/JCM.42.12.5453-5457.2004

2. Schouls LM, van der Heide HG, Vauterin L, Vauterin P, Mooi FR. Multiple-locus variable-number tandem repeat analysis of Dutch *Bordetella pertussis* strains reveals rapid genetic changes with clonal expansion during the late 1990s. J Bacteriol. 2004;186:5496–505. PubMed DOI: 10.1128/JB.186.16.5496-5505.2004

3. Parkhill J, Sebaihia M, Preston A, Murphy LD, Thomson N, Harris DE, et al. Comparative analysis of the genome sequences of *Bordetella pertussis, Bordetella parapertussis* and *Bordetella bronchiseptica.* Nat Genet. 2003;35:32–40. PubMed DOI: 10.1038/ng1227

4. Feil EJ, Li BC, Aanensen DM, Hanage WP, Spratt BG. eBURST: inferring patterns of evolutionary descent among clusters of related bacterial genotypes from multilocus sequence typing data. J Bacteriol. 2004;186:1518–30. PubMed DOI: 10.1128/JB.186.5.1518-1530.2004

5. Pupo GM, Lan R, Reeves PR, Baverstock P. Population Genetics of *Escherichia coli* in a Natural Population of Native Australian Rats. Environ Microbiol. 2000;2:594–610. PubMed DOI: 10.1046/j.1462-2920.2000.00142.x
